# Based on HIF-1*α*/Wnt/*β*-Catenin Pathway to Explore the Effect of Qingshen Granules on Chronic Renal Failure Patients: A Randomized Controlled Trial

**DOI:** 10.1155/2019/7656105

**Published:** 2019-07-02

**Authors:** Yiping Wang, Lei Zhang, Hua Jin, Dong Wang

**Affiliations:** ^1^Department of Nephrology, The First Affiliated Hospital of Anhui University of Chinese Medicine, Hefei 230031, China; ^2^Graduated School of Anhui University of Chinese Medicine, Hefei 230038, China

## Abstract

*Objectives. *This study investigates the effect of Qingshen Granules (QSG) on chronic renal failure patients and the HIF-1*α*/Wnt/*β*-catenin signaling pathway.* Methods.* Subjects were randomly divided into treatment and control groups, with 42 patients in each group. Participants in the treatment group received 10 g oral doses of QSG 3 times a day, for 12 weeks, whereas subjects in the control group were given a placebo. The effective rates of traditional Chinese medicine (TCM) symptom, serum creatinine (Scr), and estimate glomerular filtration rate (eGFR) as well as the serum levels of HIF-1*α*, Wnt1, *β*-catenin, *α*-SMA, and E-cadherin were evaluated.* Results. *Eighty patients completed the treatment program and two dropped out. After 12 weeks, the effective rates of TCM symptom and eGFR were found to be higher in the treatment group than in the control group, with statistically significant differences (*P = 0.024 *and* 0.019, *respectively). Meanwhile, lower levels of HIF-1*α*, Wnt1, *β*-catenin, *α*-SMA, and E-cadherin were detected in the treatment group, and the differences were statistically significant (*P ≤ *0.001,* P = *0.001,* P ≤ *0.001,* P ≤ *0.001, and* P = *0.039). No adverse events occurred during the study.* Conclusions.* QSG can alleviate the clinical symptoms of chronic renal failure (CRF) and protect renal function in patients by influencing the HIF-1*α*/Wnt/*β*-catenin signaling pathway. The treatment exhibits no adverse effects and is thus safe to be used by humans.

## 1. Introduction

Chronic kidney diseases (CKD) inevitably lead to chronic renal failure (CRF), a condition that is irreversible. Patients suffering from CRF rely on lifelong dialysis or kidney transplant to maintain life; otherwise, they enter the end-stage renal disease (ESRD) and may eventually die. As evidenced by national surveys in developed and developing countries, the morbidity rate of CKD is high. CKD has a high global prevalence with a consistent estimated global CKD prevalence of between 11 and 13% [1]. For example, in the United States and China, CKD morbidity rates as high as 10.2% and 10.8% are reported, respectively [2, 3]. Once CKD develops into ESRD, renal replacement therapy, an expensive treatment that is not commonly available, should be adopted. According to the annual report of the United States Renal Disease Data System (USRDS), 726,331 cases of ESRD were reported in the United States in 2018 (2206/10^6^), and medical expenditure on ESRD prevention and treatment had increased from $16.8 billion in 2003 to $35.3 billion in 2018 [4]. Renal fibrosis is a common outcome of the pathophysiological progress of CKD. Whatever their protopathy, chronic kidney diseases will eventually progress to renal fibrosis and end-stage renal failure via chronic hypoxia [5]. Overactivation of HIF-1*α* can trigger the development of renal interstitial fibrosis, which plays a vital role in hypoxia-induced renal injury [6]. The Wnt/*β*-catenin signaling pathway also contributes to the onset and progress of fibrosis* in vivo *[7]. In fact, HIF-1*α* activates the Wnt/*β*-catenin signaling pathway, which means that the latter is also involved in hypoxic pathological damage of organs [8].

At present, the means of controlling the progress of CKD and the occurrence of ESRD in modern medicine are limited and not highly effective, which has led some patients to seek complementary and alternative medicine, such as traditional Chinese medicine (TCM), for treatment [9]. Therefore, TCM has attracted the attention of many scholars who are interested in analyzing its CKD prevention and healing potential. An epidemiological study of the effect of TCM on CRF shows that spleen-kidney Qi deficiency is the most common condition in Zheng-deficiency patients and that damp-heat and blood stasis are the main pathogenic syndromes [10]. The clinical symptoms of CRF patients often manifest damp-heat syndrome. In fact, it has been confirmed that damp-heat is closely related to the occurrence and development of CRF [11]. Furthermore, the administration of TCM to CRF patients suffering from the syndrome clears away the heat and dampness, as well as removing blood stasis [12]. These results were used by the Pharmaceutical Formulations Center of the First Affiliated Hospital of Anhui University of Chinese Medicine to develop the Qingshen Granule (QSG) for the treatment of blood stasis and damp-heat syndromes. The prepared formula consists of* Herba Hedyotidis Diffusae, Rhizoma Coptidis, Herba Artemisiae Scopariae, Radix et Rhizoma Rhei, Semen Coicis, Rhizoma Atractylodis Macrocephalae, Semen Lablab Album, Poria, Radix Salviae Miltiorrhizae, Herba Leonuri, Rhizoma Alismatis, Polyporus, Herba Plantaginis, and Fructus Amomi Rotundus. *The efficiency of QSG in curing damp-heat syndrome CRF patients was tested and confirmed in a clinical study [13]. The effect of QSG was also evaluated in UUO rats, and it was shown that this medication inhibits transdifferentiation of renal tubular epithelial cells, enhances renal fibrosis resistance, and improves renal function by depressing the activity of the Wnt/*β*-catenin signaling pathway [14]. However, the effect of QSG on the pathological process of renal interstitial fibrosis induced by chronic hypoxia is unclear. Therefore, this study was conducted to explore whether QSG could inhibit the activation of HIF-1*α*/Wnt/*β*-catenin pathway in damp-heat syndrome CRF patients.

## 2. Materials and Methods

### 2.1. Trial Design

A single-blind, randomized controlled trial was conducted. After base line measurements, the patients were randomly allocated to two groups, the control group, and the treatment group, using computer-generated random numbers at 1:1 ratio. The protocols used in this study were approved by the First Affiliated Hospital of Anhui University of Chinese Medicine Ethics Committee (Hefei, China) (Approval ID: 2017AH-05) and are registered on the Chinese Clinical Trial Registry (ChiCTR-INR-17011057).

### 2.2. Sample Size

The sample size was calculated using Statistic Package for Social Science (SPSS) statistical software (version 17.0, SPSS Inc., Chicago, IL, USA) with an equal number of patients in each group (two-tailed test, power = 0.80, *α* = 0.05). The expected dropout rate was 20%. According to a previous study [13], the effective rates of TCM in control and treatment groups are assumed to be 57% and 86%, respectively, which leads to an estimated sample size of 41 patients in each group.

### 2.3. Diagnosis Criteria

The diagnostic criteria used in this study are based on the clinical practice guidelines published by Kidney Disease: Improving Global Outcomes (KDIGO) in 2012 for the evaluation and management of CKD [15]. Meanwhile, the diagnostic criteria of damp-heat syndrome are taken from the guidelines for clinical research of Chinese medicine (New Drug) [16], and they include nausea, vomiting, fatigue, poor appetite, thirst, bitter taste, abdominal distention, and sticky slimy sensation in the mouth, as well as yellow and greasy coating of the tongue. Patients manifesting yellow and greasy coating of the tongue along with at least three other symptoms are positively diagnosed with damp-heat syndrome.

### 2.4. Inclusion Criteria

To be included in the study, patients had to conform to a set of specific conditions: (1) be aged between 18 and 70 years old; (2) be diagnosed with chronic kidney disease (CKD) (3 to 5 criteria) without renal replacement therapy; (3) be diagnosed with damp-heat syndrome of TCM; (4) have blood pressure less than 140/90 mmHg, potassium level less than 5.5 mmol/L, and hemoglobin more than 80 g/L; (5) be infection-controlled, and in a stable state of illness for more than 2 weeks; and (6) have signed informed consent.

### 2.5. Exclusion Criteria

Patients with one or more of the following criteria were excluded from the study: (1) pregnant and lactating patients; (2) patients who are unable to cooperate, such as a psychotic patients; (3) patients with active malignant tumors, liver cirrhosis, decompensated or hematopoietic systems, or other serious primary diseases; (4) patients suffering from infectious diseases, acute urinary tract obstruction, or surgical treatment; (5) patients with severe arrhythmia or acute heart failure (grade NYHA or above. New York Heart Association grades III and IV), or those suffering from myocardial infarction or cerebrovascular events for less than 3 months; (6) patients diagnosed with diabetic nephropathy; (7) patients using corticosteroids, nonsteroidal anti-inflammatory drugs, or immunosuppressive agents; (8) patients who are known to be allergic to certain drugs used in this study; and (9) patients participating in other clinical trials.

### 2.6. Exit Criteria

Participants who suffered from severe liver or kidney dysfunction, malignancy, psychiatric disorders, acute cardiocerebrovascular events within the last six months, complications of other endocrine diseases, or serious primary diseases were also excluded.

### 2.7. Intervention

The basic treatment regimen covers nutritional therapy and blood pressure control, as well as treatment of anemia and mineral or bone disorders. The therapeutic goal was set according to the guidelines published in KDIGO. Participants in the treatment group received 10 g oral doses of QSG (offered by the Pharmaceutical Formulations Center of the First Affiliated Hospital of Anhui University of Traditional Chinese Medicine, Anhui, China [Lot, BZ20080011]) three times a day, whereas patients in the control group were given a similar dosage of a placebo containing 5% QSG. The shape, color, and packaging of the placebo were consistent with those of QSG. The duration of the treatment program was set to 12 weeks.

### 2.8. Outcome Measures

The primary outcomes are the levels of serum creatinine (Scr), the glomerular filtration rate (eGFR calculated by CKD-EPI formula), and the effective rate of TCM symptom [16] (divided into four grades: significantly effective, effective, stable, and invalid). Other results include levels of serum HIF-1*α* (ELISA Kit, Elabscience, Batch number: E-EL-H1277), Wnt1 (ELISA Kit, Elabscience, Batch number: SEL821Hu), *β*-catenin (ELISA Kit, Elabscience, Batch number: E-EL-H0666c), *α*-SMA (ELISA Kit, Elabscience, Batch number: E-EL-H0979c), and E-cadherin (ELISA Kit, Elabscience, Batch number: E-EL-H0014c).

### 2.9. Safety Assessments

An adverse event is defined as any untoward medical occurrence that may present itself during the course of the study and that may or may not be instigated by the procedures performed therein. A serious adverse event is an adverse event resulting in death, a life-threatening experience, hospitalization, or significant disability/incapacity [17]. All such events were recorded in detail throughout the study and related using medical terminology. Severe adverse events were also reported to the institutional review board and/or independent ethics committees, as well as to the principal investigator, within 24 hours.

### 2.10. Statistical Analysis

Statistical analysis was performed using the software package SPSS 20.0 (SPSS, Inc., USA). Normally distributed measurement data were expressed as means and standard deviations (M ± SD). The effective rates in different groups were compared using the Chi-square test, whereas differences before and after treatment were evaluated by paired t-test. The ranked data were assessed using the Ridit test.* P* < 0.05 was considered to be statistically significant.

## 3. Results

**Figure 1:**
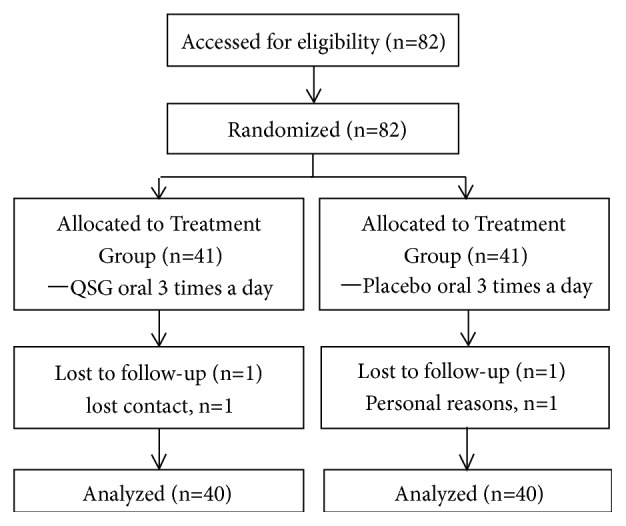
Study flow diagram.

Eighty-two CKD patients with damp-heat syndrome were included in the study and randomly assigned to treatment or control groups. Eighty out of the eighty-two were able to complete the study, and two dropped out (one in each group) (Figure 1). Baseline characteristics for all groups are shown in Table 1. No significant differences were detected among the groups at baseline, and the two groups are comparable.

*Comparison of the effective rates of TCM symptom.* After 12 weeks of treatment, the effective rate of TCM symptom ((significantly effective + effective + stable)/40) was found to be 80% and 60% in the treatment and control groups, respectively. Thus, higher rates were observed with QSG treatment (*P = 0.024*) (Table 2).

*Comparison of the levels of Scr and eGFR. *Before the administration of QSG, there were no statistically significant differences between the two groups in terms of Scr (*t =*-0.133,* P = *0.859) and eGFR (*t =*-0.244,* P = *0.823) levels. After 12-week treatment, the levels of Scr and eGFR in both groups were decreased, with the amount of Scr in the treatment group being lower than that in the control group, but without statically significant differences (*t =*-1.602,* P = *0.113). Meanwhile, the level of eGFR was higher in the treatment group as compared with the control group and with statically significant differences (*t = *2.388,* P = *0.019), (Table 3).

*Comparison of the levels of HIF-1α, Wnt1, β-catenin, α-SMA, and E-cadherin. *Before treatment, there were no statically significant differences between the two groups when comparing the levels of HIF-1*α* (*t =*-0.672,* P = *0.504), Wnt1 (*t =*-0.224,* P = *0.823), *β*-catenin (*t = *0.266,* P = *0.719), *α*-SMA (*t =*-0.279,* P = *0.781), and E-cadherin (*t = *0.013,* P = *0.897). At the end of the 12-week treatment program, the levels of HIF-1*α*, Wnt1, *β*-catenin, *α*-SMA, and E-cadherin in both groups were decreased, with the treatment group showing lower concentrations than the control group (*P ≤ *0.001*, P = *0.001*, P = *0.001*, P ≤ *0.001*, P ≤ *0.001, and* P = *0.039) (Table 4).

*Adverse events.* No adverse events occurred.

## 4. Discussion

The final outcome of pathological changes in CRF is renal interstitial fibrosis, which is mainly characterized by the proliferation of fibroblasts and deposition of extracellular matrix. Epithelial-Mesenchymal Transition (EMT) plays an important role in the pathological evolution of renal interstitial fibrosis, as it refers to the transformation of renal tubular epithelial cells to myofibroblasts by various pathological factors. It is also involved in the growth of the extracellular matrix, which is the key reason for the deterioration of renal function [18]. Extensive research in modern medicine has been conducted to produce effective cures for renal fibrosis. However, the rates of recovery are still limited, due to the scarcity of efficient drugs. TCM is one treatment that can successfully delay the progress of renal fibrosis for a long time. Studies confirm that QSG, a Chinese herbal formula, significantly improves the clinical symptoms and life quality of CRF patients and even reduces the levels of many inflammatory factors [19, 20]. At the same time, studies conducted on UUO rats show that it inhibits the activation of many signal pathways, such as NF-kappa B [21] and JAK/STAT [22], thereby impeding fibrosis. *α*-SMA is a marker protein of EMT. During the progression of renal fibrosis, glomerular parietal epithelial, endothelial, mesangial, and tubulointerstitial cells transdifferentiate into myofibroblasts to express *α*-SMA, which indirectly reflects the degree of renal interstitial fibrosis [23]. E-cadherin is an important intercellular adhesion molecule that exists in the form of a transmembrane glycoprotein on the cell surface. It is mainly found in renal tubular epithelial cells of normal kidney tissue where it plays a role in mediating adhesion reactions between homologous cells, maintaining cell polarity, and regulating differentiation, all of which are important for the preservation of tissue morphology and structure [24]. When EMT occurs, intercellular adhesion and epithelial protein levels decrease, as evidenced by the reduced expression of E-cadherin in tissues, compared to increased levels of this protein in serum. The main reason behind this is that, during the course of fibrosis, E-cadherin in tissues is completely degraded and released into serum to form soluble E-cadherin [25]. This study shows that QSG is capable of delaying the progress of renal dysfunction in CRF patients with damp-heat syndrome by reducing the levels of *α*-SMA and E-cadherin in peripheral blood and regulating the levels of HIF-1*α*, Wnt1, and *β*-catenin in serum.

Chronic hypoxia plays a key role in the instigation and progression of renal interstitial fibrosis [5, 26]. In the early stages of CKD, the oxygen supply in kidney tissues is diminished, resulting in high energy consumption of renal tubular epithelial cells, which renders the kidney vulnerable to ischemia and hypoxia injury. Hypoxic renal injury is mainly mediated by HIF-1, which is the only specific transcription factor that can exert biological activity under hypoxia conditions [27]. The overexpression of HIF-1 and its main active subunit, HIF-1*α*, in renal tubular cells leads to the development of renal fibrosis when hypoxia occurs [28, 29]. During hypoxia, p53 upregulation induced by HIF-1*α* suppresses cell cycle progression, leading to the accumulation of G2/M cells, and activates profibrotic TGF-*β* and CTGF-mediated signaling pathways, causing extracellular matrix production and renal fibrosis [30]. In this study, we show that QSG is capable of reducing HIF-1*α* levels in CRF patients, thereby limiting the progression of renal fibrosis.

Some studies have shown that the increase in HIF-1*α* expressions and the development of renal fibrosis are related to the activation of the Wnt/*β*-catein signaling pathway [31, 32], which is caused by podocyte dysfunction and promotes the formation of proteinuria [33]. Wnt is a highly conserved signaling pathway that widely exists in eukaryotic organisms [34]. It plays an important role in the development of organisms and participates in the process of cell proliferation, differentiation and apoptosis. Overexpression of Wnt1* in vivo* activates the expression of *β*-catenin in glomeruli and accelerated the formation of proteinuria, while the antagonist gene DKK1 improves podocyte injury. DKK-1 also reduces the accumulation of *β*-catenin in the kidneys of UUO rats. The expressions of col-I and FN are reduced by inhibiting the Wnt/*β*-catenin signaling pathway [35]. This study provides evidence that QSG downregulates the levels of Wnt and *β*-catenin in CRF patients.

## 5. Conclusion

The single-blind randomized controlled trials conducted in this study confirm that QSG alleviates the clinical symptoms of damp-heat syndrome in CRF patients and reduces the serum level of creatinine, HIF-1a, Wnt1, *β*-catenin, *α*-SMA, and E-cadherin. This indicates that QSG interferes with the activation of the HIF-1*α*-mediated Wnt/*β*-catenin signaling pathway. No adverse drug reactions were recorded during the intervention process, which indicates that QSG is safe.

## Figures and Tables

**Table 1:** Comparison of baseline demographic characteristics in the treatment and control groups (M ± SD).

Variable	Treatment group	Control group	*t/X* ^*2*^	*P*
Male/female	22/18	19/21	0.450	0.502
Age (year)	52.07±10.42	52.85±9.16	-0.353	0.725
Basic disease				
CGN	23	20	0.453	0.501
HN	15	17	0.208	0.648
PK	2	3	0.213	0.644
Stage				
CKD3	17	16	0.520	0.820
CKD4	13	15	0.220	0.639
CKD5	10	9	0.069	0.793

CGN: chronic glomerulonephritis, HN: hypertensive nephropathy, and PK: polycystic kidney.

**Table 2:** Comparison of the effective rates of TCM symptom.

Group	Significantly effective	Effective	Stable	Invalid	*z*	*P*
Treatment group	10	12	10	8	-2.251	0.024
Control group	6	6	12	16		

**Table 3:** Comparison of Scr and eGFR levels (M ± SD).

Variable		Treatment group	Control group	*t*	*P*
Scr (umol/L)	pre	391.2±62.9	393.3±77.8	-0.133	0.859
	post	341.8±63.2	365.9±70.7	-1.602	0.113
eGFR (ml/min)	pre	13.5±2.5	13.6±4.1	-0.244	0.823
	post	15.9±3.2	14.0±4.0	2.388	0.019

**Table 4:** Comparison of HIF-1*α*, Wnt1, *β*-catenin, *α*-SMA, and E-cadherin levels (M ± SD).

Variable		Treatment group	Control group	*t*	*P*
HIF-1*α*(ng/ml)	pre	1.71±0.33	1.76±0.31	-0.672	0.504
	post	0.66±0.16	1.39±0.17	19.846	≤0.001
Wnt1(pg/ml)	pre	378.2±88.0	382.5±85.4	-0.224	0.823
	post	314.2±85.8	382.8±85.3	-3.584	=0.001
*β*-catenin(pg/ml)	pre	462.6±13.6	461.7±16.7	0.266	0.719
	post	416.5±13.6	462.1±15.1	-14.176	≤0.001
*α*-SMA(KU/L)	pre	25.9±4.6	26.2±5.0	-0.279	0.781
	post	20.5±3.1	23.5±4.1	-3.754	≤0.001
E-cadherin(ng/ml)	pre	2553.5±404.4	2520.2±505.7	0.013	0.897
	post	2166.9±398.6	2370.7±468.0	-2.097	0.039

## Data Availability

Readers can access the data underlying the findings of the study by contacting the corresponding author at wypwyp54@aliyun.com or at the Chinese Clinical Trial Registry (ChiCTR-INR-17011057).

## Supplementary Materials

Supplementary Materials Supplementary materials which were submitted along with our manuscript were the original data of our article. TCM syndrome scores, serum creatinine, E-cadherin, *α*-SMA, HIF-1*α*, Wnt1, and *β*-catenin.
